# What Do Secure Care Stakeholders Want From the Forensic MDT? a Qualitative Study With Service Users, Carers, and Nurses

**DOI:** 10.1192/bjo.2022.228

**Published:** 2022-06-20

**Authors:** Fiona Hynes, Alexander Jack, Eleanor Parkinson, Steven Hemblade, Talhah Malik

**Affiliations:** 1 Birmingham & Solihull Mental Health NHS Foundation Trust, Birmingham, United Kingdom.; 2 University of Wolverhampton, Wolverhampton, United Kingdom; 3 Priory Hospital Burgess Hill, Burgess Hill, United Kingdom

## Abstract

**Aims:**

Clinical teams oversee the care of patients within secure psychiatric inpatient settings. They are made up of a number of professions, including psychiatrists, psychologists, occupational therapists, social workers and nurses. The effective collaboration of the different members of the clinical team is vital for its functioning. However, so is the team's interface with other key stakeholder groups, namely nursing teams, service users and carers. Understanding the needs and priorities of these groups regarding their relationships with the clinical team is also important to recognise and in the provision of good quality care. This study aims to understand the experiences, priorities and needs of stakeholder groups in their relationship with the clinical team. Gaining feedback from multiple sources (service users, carers, nurses) will help facilitate functioning of the clinical team in the delivery of excellent care to service users.

**Methods:**

Ethical approval was granted by the host NHS trust. Between October 2019 and October 2021, three focus groups were conducted using a semi-structured interview to gather responses from carers, nurses and service users (6 participants in each group) respectively. The interviews were recorded and transcribed. Thematic analysis was used to code each transcript and themes were drawn from the coded data.

**Results:**

Dominant themes emerged from the three data sets. Consistent themes between groups included communication, hierarchy/power and representation. There were also differences in themes identified, with the carer group bringing the theme of education/ knowledge, and nursing group raising the value of human relationships, including compassion. The theme of transparency emerged strongly for the service user group.

**Conclusion:**

This study offers an interesting perspective on what distinct stakeholder groups want and value in their relationship with the clinical team. Gaining feedback from multiple sources (service users, carers, nurses and members of the MDT) can better inform a team about its functioning and help improve performance. Developing a tool to aid the systematic collection of multi-source feedback is the next step of this project, facilitating the voices of key stakeholder groups to be heard.

